# Selective Expression of Nicotinic Receptor Sub-unit mRNA in Early Human Fetal Forebrain

**DOI:** 10.3389/fnmol.2020.00072

**Published:** 2020-05-21

**Authors:** Ayman Alzu’bi, William Middleham, Mohammed Shoaib, Gavin J. Clowry

**Affiliations:** ^1^Biosciences Institute, Newcastle University, Newcastle upon Tyne, United Kingdom; ^2^Department of Basic Medical Sciences, Yarmouk University, Irbid, Jordan

**Keywords:** cerebral cortex, development, ganglionic eminences, glutamatergic neurons, inhibitory interneurons, nicotine, nicotinic receptors

## Abstract

Increasing evidence from animal and human studies indicate that exposure to nicotine during development, separated from the effects of smoking tobacco, can contribute to dysregulation of brain development including behavioral deficits. An RNAseq study of human fetal cerebral cortex demonstrated that 9 out of 16 genes for human nicotinic acetylcholine (ACh) receptor subunits are selectively expressed between 7.5 and 12 post-conceptional weeks (PCW). The most highly expressed subunit genes were *CHNRA4* and *CHNRB2*, whose protein products combine to form the most ubiquitous functional receptor isoform expressed in the adult brain. They exhibited correlated expression in both RNAseq samples, and in tissue sections by *in situ* hybridization. Co-localization studies with other cortical markers suggest they are pre-dominantly expressed by post-mitotic glutamatergic neuron pre-cursors in both cortical plate and pre-subplate, rather than cortical progenitor cells or GABAergic interneuron pre-cursors. However, GABAergic interneuron progenitor cells in the ganglionic eminences do express these sub-units. *CHNRA5* also showed moderate levels of expression and again favored post-mitotic neurons. Other subunits, e.g., *CHRNA7*, exhibited low but detectable levels of expression. *CHRN* genes found not to be expressed included genes for subunits usually considered muscle specific, e.g., *CHNRA1*, although some muscle specific gene expression was detected, for instance *CHNRB1*. Although there is little or no synthesis of acetylcholine by intrinsic cortical neurons, cholinergic fibers from basal forebrain innervate the cerebral cortex from 12 PCW at the latest. Acetylcholine may have a paracrine effect on radially migrating cortical neurons and GABAergic interneuron progenitors.

## Introduction

Nicotinic acetylcholine receptors (nAChRs) are pentameric ligand-gated cation channels that respond to acetylcholine, as well as a variety of pharmacological agents including nicotine (Albuquerque et al., 2009; Kulbatskii et al., 2018). They are widely distributed throughout human and rodent brain during all phases of development (Broide et al., 1995; Zoli et al., 1995; Agulhon et al., 1999; Hellstrom-Lindahl and Court, 2000; Pentel et al., 2006; Broide et al., 2019). Depending on their subunit composition, nAChRs can gate both Na^+^ and Ca^++^ ions and exist in one of three conformational states: open, closed at rest, and desensitized in which ligand binding cannot induce channel opening (Dani and Bertrand, 2007). There are up to 12 genes (species dependent) that encode the subunit proteins believed to be expressed in the central and autonomic nervous system, yielding seven α (α2–α10) and three β subunits (β2–β4) (McGehee, 1999). These combine in both homomeric (α7–α10) and heteromeric (α2–α7, β2–β4) configurations, which determine the pharmacological specificity, ion selectivity, and desensitization characteristics of the nAChR (Gotti et al., 2009; Wu et al., 2016). Other subunits are expressed at the neuromuscular junction in different configurations depending on the development stage (α1, αδ, αγ, αε, and β2; Cordero-Erausquin et al., 2000) and heteromeric combinations of α9α10 subunits may be expressed in mammalian vestibular and cochlear mechanosensory hair cells (Elgoyhen et al., 2001).

Tobacco smoke contains a complex mixture of many chemicals which may be potentially interfere with fetal development (Hoffmann and Hoffmann, 1997; Pazo et al., 2016). In particular, carbon monoxide can reduce oxygen transport across the placenta leading to hypoxia and growth retardation (Verhagen et al., 2011) particularly in the third trimester, which may have specific effects on neural systems, for instance dopaminergic innervation of the prefrontal cortex, leading to attention deficit and hyperactivity disorder (Zhu et al., 2016). For these reasons, nicotine replacement therapy has been proposed as advisable for pregnant women who find it hard to stop smoking during pregnancy (Benowitz and Dempsey, 2004). A recent study has shown that nicotine replacement therapy may be relatively protective of the fetus, compared to smoking, for those births that reach full term (Tran et al., 2020). Nevertheless nicotine, which is the major psychoactive component of tobacco smoke and the primary cause of addiction, has been shown to cross the placenta, enter fetal circulation and accumulate in the fetus from as early as 7 weeks of gestation (Jauniaux et al., 1999). A large number of studies have shown a correlation between maternal smoking during pregnancy and psychiatric disorders during later life (e.g., Mansi et al., 2007; Wehby et al., 2011; Ekblad et al., 2014; Dong et al., 2018) while experiments in animals have postulated potential roles for both nicotine exposure (Role and Berg, 1996; Pilarski et al., 2012; Wang and Gondre-Lewis, 2013; Zhang et al., 2019) and perinatal hypoxia (Miguel et al., 2015, 2018) in affecting neurodevelopment leading to psychiatric disease.

Interestingly, Dong et al. (2018) provided evidence that even after stopping in the first trimester smoking was still significantly associated with childhood ADHD, suggesting that the first trimester could be an important window for fetal neurodevelopment during which exposure to smoking could be a risk factor for childhood ADHD. At this early stage of development mild hypoxia is likely to be less of a threat to cortical development (Jaddoe et al., 2007). In support if this, it has been shown that third trimester smoking has deleterious effects upon growth rates, whereas smoking throughout pregnancy affects attention in neonates (Espy et al., 2011). In animal experiments, developmental exposure to nicotine causes changes in both neuronal structure and behavior (Sorenson et al., 1991; Heath and Picciotto, 2009; Ballesteros-Yanez et al., 2010; Lozada et al., 2012; Mychasiuk et al., 2013) and may act on gene expression that controls cortical circuit formation via the epigenetic regulator *Ash2l*, a component of a histone methyltransferase complex (Jung et al., 2016). It has been demonstrated that in the brainstem and cerebellum of the human fetus between 5 and 12 weeks gestation the gene expression pattern of both α4 and α7 nicotinic receptor subunits was different following smoking during the pregnancy (Falk et al., 2005).

Therefore, we propose that nicotinic receptors may be expressed in the human forebrain during the early stages of development, such that exposure to nicotine could disturb the very earliest stages of cortical circuit formation. Previous studies have demonstrated that nicotinic receptor sub-units are expressed in human fetal forebrain extracts at both the mRNA and protein level from about 8 post-conceptional weeks (PCW) by RT-PCR and the binding of a radioligand to membrane fractions (Hellstrom-Lindahl et al., 1998; Falk et al., 2002) and from 17 PCW by immunohistochemistry and *in situ* hybridization for the α4 subunit only (Schröder et al., 2001). However, to date, no studies have attempted to localize receptor expression to specific cell types in the developing human forebrain. This study presents a comprehensive exploration of nicotinic receptor subunit expression by RNAseq, coupled with *in situ* hybridization and immunohistochemical approaches to elucidate cell specific expression.

## Materials and Methods

### Human Tissue

Human fetal tissue from terminated pregnancies was obtained from the joint MRC/Wellcome Trust-funded Human Developmental Biology Resource (HDBR)^1^ (Gerrelli et al., 2015). All tissue was collected with appropriate maternal consent and approval from the Newcastle and North Tyneside NHS Health Authority Joint Ethics Committee. Fetal samples ranging in age from 7.5 to 12 PCW were used. Ages were estimated from foot and heel to knee length measurements according to Hern (1984).

For RNAseq, whole fetal brains were isolated from the skull and the meninges were removed. The hemispheres were separated and the choroid plexus and subcortical structures removed. One or both hemispheres was then divided into a maximum of 7 sections. Each hemisphere represented an independent sample. The temporal lobe, including lateral and medial walls was removed, and divided in half and labeled section 6 (posterior temporal cortex) or 7 (anterior). The remaining cortex was divided into 5 sections of equal width from the anterior (A) to the posterior (P) pole of the cortex including lateral and medial cortical walls (labeled 1–5) depending on size. RNA was extracted from the sections. Each RNA sample was described by age in PCW, and by position by giving an average numerical value derived by considering the anterior pole as 0, posterior pole as 5 and the temporal pole as 7 (after Miller et al., 2014). Therefore for a hemisphere minus temporal lobe divided into 5, slice 1 has a locational average value of 0.5, whereas slice 3 has a value of 2.5, slice 5 has a value of 4.5 and slice 7, 6.5, etc.

For *in situ* hybridization, brains were isolated and fixed for at least 24 h at 4 °C in 4% paraformaldehyde (Sigma-Aldrich, Poole, United Kingdom United Kingdom) dissolved in 0.1 M phosphate-buffered saline (PBS). Once fixed, whole or half brains (divided sagittally) were dehydrated in a series of graded ethanols before embedding in paraffin. Brain samples from fetuses aged 8, 10, and 12 PCW were cut at 8 μM section thickness in one of three different planes; horizontally, sagittally, and coronally, and mounted on slides.

### RNA Seq

Full details of the origins, collection, preparation, sequencing and analysis of the human fetal RNA samples has been previously described (Lindsay et al., 2016; Harkin et al., 2017). The entire RNAseq dataset from which data was extracted for this study has been deposited at www.ebi.ac.uk/arrayexpress/experiments/E-MTAB-4840. High quality reads were then mapped to the human reference genome hg38 with Tophat2 (Kim et al., 2013). Reads aligned to genes and exons were counted with htseq-count (Anders et al., 2014) and normalized RPKMs calculated. Read length was 101 bp prior to trimming and 85 bp after trimming with no reads of less than 20 bp retained. The minimum number of reads examined per sample was 63 million (average 90 million).

### RNAScope *in situ* Hybridization

RNA *in situ* hybridization experiments were performed using the RNAscope^®^ technology, which has been previously described (Wang et al., 2012). Paired double-Z oligonucleotide probes were designed against target RNA using custom software. The following probes were used:- *Hs-CHRNA4* (498331) a 20zz probe targeting 2,085–3,748 of NM_000744.6: *Hs-CHRNA5* (482401) a 20zz probe targeting 282–3,587 of NM_000745.3; Hs-*CHRNA7* (310101) a 21zz probe targeting 181–1,591 of NM_000746.5; Hs-*CHRNB2* (498351) a 20zz probe targeting 502–2,170 of NM_000748.2. The *RNAscope* Reagent Kit (ACD Bio Techne, Abingdon, United Kingdom) was used according to the manufacturer’s instructions but with slight modifications. In brief, 8 μM−thick paraffin sections were baked on a heating pad for 10 min at 60°C, dewaxed in Xylene, and then boiled with target retrieval buffer (ACD) for 20 min at 95°C. Protease digestion was carried out at 40°C for 30 min, followed by probe hybridization for 2 h at 40°C with target probes. The hybridized signals were amplified by a cascade of signal amplification molecules and detected with the RNA*scope* 2.5 HD detection kit (Fast Red). Slides were counterstained with 50% hematoxylin and positive signals showed as red chromogenic dots in the cytoplasm or nucleus. Each sample was quality controlled for RNA integrity with a probe specific to the housekeeping gene *GAPDH*. Negative control background staining was evaluated using a probe specific to the bacterial *dapB* gene.

### RNAscope Fluorescent *in-situ* Hybridization Coupled With Immunofluorescence

*In situ* hybridization was carried out first as described above using probes for human *CHRNA4* and *CHRNB2* only. Instead of Fast Red, the hybridized signals were detected with Cy3 tyramide (Tyramide Signal Amplification (TSA^TM^) Cy3 plus system reagent, Perkin Elmer, Buckingham, United Kingdom). Then immunofluorescent staining was carried out according to previously described protocols (Alzu’bi et al., 2017) with antibodies to either GAD67 (interneuron marker; mouse monoclonal dilution 1:1000, Merck Millipore, Watford, United Kingdom; AB_2278725; Alzu’bi and Clowry, 2019) or Ki67 (progenitor cell marker; mouse monoclonal dilution 1:150; Dako, Ely, United Kingdom; AB_2142378; Alzu’bi and Clowry, 2019). Briefly, sections were boiled in 10 mM citrate buffer pH6, followed by incubation with primary antibody [diluted in 10% normal blocking serum in Tris buffered saline (TBS) pH 7.6] overnight at 4^0^C. Sections were then incubated with HRP-conjugated secondary antibody for 30 min [ImmPRESS^TM^ HRP IgG (Peroxidase) Polymer Detection Kit, Vector Labs]. Signals were detected with fluorescein tyramide for 10 min (TSA^TM^ fluorescein plus system reagent, Perkin Elmer). Sections were counterstained with 4’, 6-diamidino-2-phenylindole dihydrochloride (DAPI; Thermo Fisher Scientific, Cramlington, United Kingdom) and mounted using Vectashield Hardset Mounting Medium (Vector Labs, Peterborough, United Kingdom).

## Results

### Quantitative Analysis of mRNA Reveals Distinct Patterns of *CHNR* Gene Expression

**FIGURE 1:**
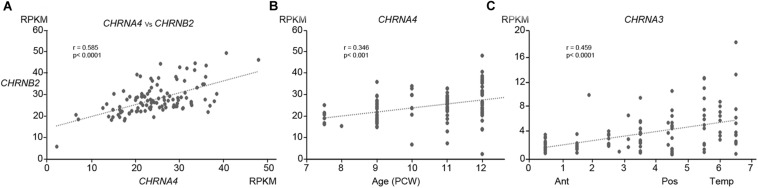
Patterns of expression of nAChR subunit mRNA. **(A)** Shows the high correlation of expression (normalized RPKM) of *CHRNA4* with *CHRNB2* within specific tissue samples. **(B)** Shows that expression of CHRNA4 increased significantly with age (PCW, post-conceptional weeks) but only to a small degree, while **(C)** demonstrates that expression of CHRNA3 changed significantly but only to a small degree across cortical regions (ant, anterior; Pos, Posterior; Temp, temporal). **(B,C)** Are representative of the degree to which other subunits change expression over time or across the cortex (see Table 1).

One hundred and nine samples of cortical tissue taken from multiple locations across the cortical surface at ages ranging from 7.5 to 12 PCW, were subjected to RNAseq analysis. Of the sixteen nicotinic receptor sub-unit genes (*CHNR*) present in the human genome, nine were expressed at this stage of development, of which four could be regarded as moderately expressed (in the second quartile) and four showed low levels of expression (third quartile) in comparison to expression of all protein coding genes at this stage of development (see Table 1 and Harkin et al., 2017, for more details of expression level determination). Surprisingly, expression of two subunits, *CHNRB1* (moderate expression) and *CHNRE* (low) usually associated with the neuromuscular junction rather neuron-neuron synapses (Cordero-Erausquin et al., 2000) was detected. Interestingly, although *CHNRB1* is not thought to be expressed in the brain, whole genome linkage analysis has found an association with nicotine dependence (Lou et al., 2007). The most highly expressed genes were *CHRNA4* and *CHRNB2*, the protein products of which together form the most ubiquitous isoform of the nicotinic receptor in the adult cerebral cortex and thalamus (α4_2_β2_3_; Cordero-Erausquin et al., 2000). Expression of the two subunits was highly correlated between samples (Figure 1A) providing indirect evidence that they were co-expressed to form this receptor sub-type. Of the other subunit genes usually pre-dominantly expressed in the central nervous system (Cordero-Erausquin et al., 2000) *CHRNA5* was moderately expressed and *CHRNA7* and *CHRNA10* exhibited low levels of expression. *CHRNFAM7A*, a partial duplication of the *CHRNA7* gene which codes for a non-acetyl choline binding dupα7 subunit that assembles with α7 subunits, resulting in a dominant negative regulation of receptor function (Sinkus et al., 2015), was found to exhibit very low levels of expression.

**TABLE 1:** Summary of expression of nicotinic receptor subunits in the human fetal cerebral cortex.

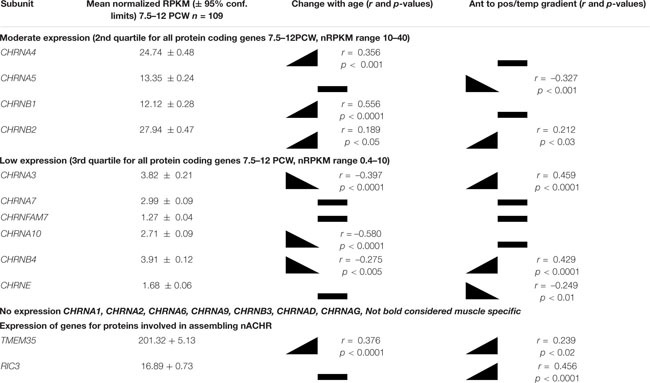

Expression for all studied genes changed relatively little with either age or location within the cortex, although small but statistically significant differences were detected for some genes (Table 1 and Figures 1B,C). Apart from *CHNRA5*, all moderately expressed genes significantly increased expression with age, whereas genes exhibiting low expression were either unchanged or showed a slight tendency to decrease expression. Gene expression levels for a number of *CHNR* genes did show a tendency to correlate with cortical location which was given a numerical value derived by considering the anterior pole as 0, posterior pole as 5 and the temporal pole as 7 (see methods; Miller et al., 2014) showed that many genes were expressed in gradients along this axis. *CHNRA3* (Figure 1C; see also Lindsay et al., 2016) and *CHNRB4* both showed a strong tendency to increase expression toward the posterior and temporal cortical areas, as did *CHNRB2* to a lesser extent (Table 1). A 3_2_β4_3_ isoforms of the nicotinic receptor are usually associated with autonomic ganglia and certain subcortical brain nuclei (Gotti et al., 2009) and so it is speculative to suggest such isoforms might be present in the developing cerebral cortex. *CHRNA5* and *CHRNE* were both slightly more highly expressed in the frontal cortex.

In order for nicotinic receptor subunits to assemble into functional receptors, expression of other proteins is required. Two examples of this are NACHO (gene name *TMEM35*) and RIC3 (Koperniak et al., 2013; Gu et al., 2016; Matta et al., 2017). Therefore expression of these genes was also explored. It was found that *TMEM35* is very highly expressed whilst *RIC3* is also expressed at similar levels to *CHRNA* genes (see Table 1). Therefore, it is a strong possibility that functional receptors are formed in the early fetal cortex.

### *In situ* Hybridization

**FIGURE 2:**
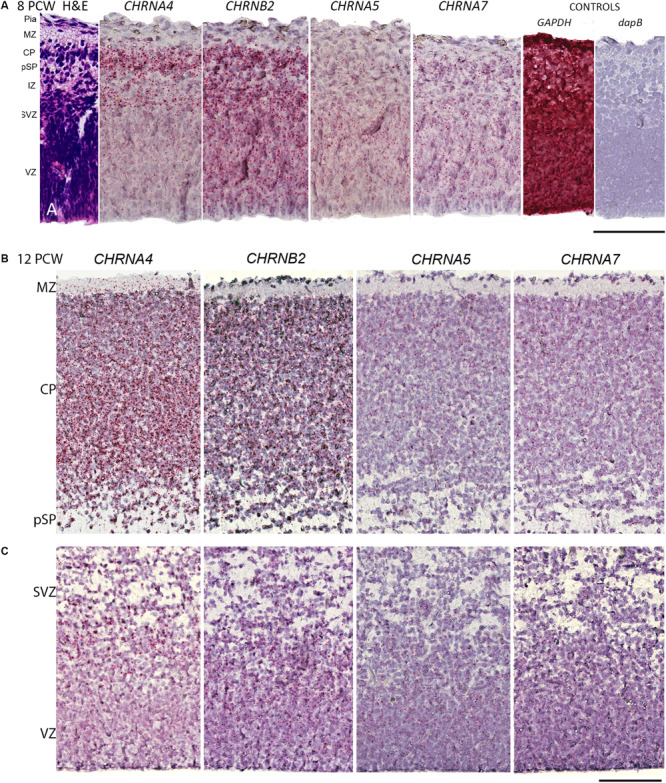
RNAScope *in situ* hybridization for nAChR subunit mRNA. **(A)** Sections from across the whole dorsomedial cortical wall at 8 post-conceptional weeks (PCW) with positive *in situ* hybridization observed as red dots and counterstained with hemotoxylin. *CHRNA4* was expressed pre-dominantly in layers containing post-mitotic cells, *CHRNB2* was strongly expressed in all compartments, *CHRNA5* showed lower expression which was slightly higher in proliferative layers, whereas *CHRNA7* was weakly expressed. Probes for the standard reference gene *GAPDH* detected very strong expression, whereas the bacterial gene *dapB* showed no detectable expression. **(B,C)** A broadly similar pattern of expression was seen at 12 PCW, but with *CHRNA4* now all but absent from the purely proliferative VZ, and *CHRNA5* expression slightly stronger in the CP. MZ, marginal zone; CP, cortical plate; pSP, pre-subplate; IZ, intermediate zone; SVZ, subventricular zone; ventricular zone. Scale bars = 100 μM.

**FIGURE 3:**
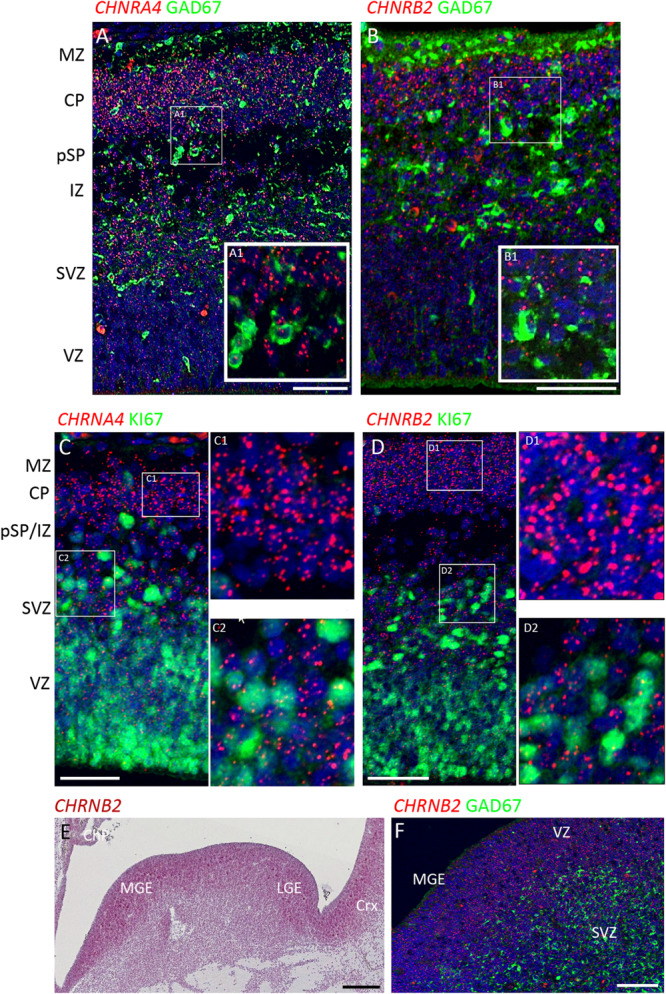
Combined *in situ* hybridization/immunohistochemistry to localize nAChR subunit expression to cell type. **(A,B)** Higher magnification insets A1, B1, show combined expression of either *CHRNA4* or *CHRNB2* (red) with GAD67 (green) a marker for inhibitory interneurons. Sections were counterstained with DAPI (blue). Although both sub-unit mRNAs were strongly expressed in post-mitotic zones, there was little co-localization with GAD67 suggesting their expression may very largely be confined to glutamatergic neurons. Conversely, in proliferative zones (**C,D** and higher magnification insets C1,2 and D1,2) *CHRNA4* and *CHRNB2* expression was generally not co-localized to cells expressing KI67, a marker for dividing cells. In the ventral telencephalon, *CHRNB2* **(E)** and *CHRNA4* (not shown) were strongly expressed in the proliferative ganglionic eminences, particularly in the MGE, a source of cortical interneurons. However, again, neither *CHRNB2* **(E)** *n*or *CHRNA4* (not shown) was observed to co-localize with GAD67 in post-mitotic cortical interneuron pre-cursors **(F)**. MZ, marginal zone; CP, cortical plate; pSP, pre-subplate; IZ, intermediate zone; SVZ, subventricular zone; VZ, ventricular zone; ChP, choroid plexus; MGE, medial ganglionic eminence; LGE, lateral ganglionic eminence; Crx, cerebral cortex. Scale bars = 100 μM.

Initially, immunohistochemistry for nicotinic receptor subunits was attempted using various commercially available antibodies but these proved unreliable, as previously reported (Moser et al., 2007) and so are not reported here. Instead we turned to RNAscope *in situ* hybridization to validate our RNAseq findings and found this to be satisfactory. The protein products of *CHNRA4* and *CHRNB2* are known combine to form a functioning receptor isoform in the brain (α4_2_β2_3_) sometimes incorporating an α5 subunit from the *CHRNA5* gene (α4_2_β2_2_α5) which increases the magnitude of nicotine-gated currents (Bailey et al., 2010). For the two most highly expressed genes, *CHNRA4* and *CHNRB2*, expression levels were higher in the post-mitotic layers (marginal zone, cortical plate, pre-subplate) of the cortical wall than in the proliferative layers (ventricular zone, subventricular zone) at all ages studied, especially for *CHNRA4* (Figures 2A–C, 3A–D). *CHNRA5* showed a lower level of expression (Figures 2A–C) reflecting the RNAseq findings. At 12 PCW there was a tendency toward higher expression in neurons in the outer layers of the cortical plate (recently arrived) than in lower levels (born earlier; Figure 2B). Low level *CHNRA7* expression was detected by *in situ* hybridization and was located throughout the cortical wall at all ages studied (Figures 2A–C) confirming our RNAseq findings (Table 1) and a previous *in situ* hybridization study (Agulhon et al., 1999). The probes might have detected *CHRFAM7A* expression as well, due to the high homology between these genes in the probe target region.

To further characterize expression the two most highly expressed sub-units, double label fluorescent *In situ* and immunofluorescence was carried out for *CHNRA4* and *CHNRB2* with either GAD67 (marker for GABAergic interneurons) or KI67 (marker for dividing cells) at 8 PCW. It was found that neither *CHNRA4* nor *CHNRB2* co-localized with GAD67+ neurons to any great extent, instead being preferentially expressed by GAD67- cells in the post-mitotic layers of the cortex, which are pre-dominantly glutamatergic pyramidal cell pre-cursors either migrating through the intermediate zone or settled in the cortical plate and pre-subplate (Figures 3A,B). Similarly, in the proliferative zones, low levels of *CHNRA4* or *CHNRB2* expression showed little co-localization with KI67+ cells undergoing cell division and preferentially localized to KI67- cells, which could either be quiescent progenitors not undergoing division, or newborn post-mitotic neuron precursors (Figures 3C,D). Interestingly, in the ganglionic eminences of the ventral telencephalon, where cortical GABAergic interneurons and GABAergic neurons of the basal ganglia are born (Marin and Rubenstein, 2001; Alzu’bi et al., 2017) high expression of both *CHNRA4* (not shown) and *CHNRB2* (Figures 3E,F) was observed, co-localized with progenitor cells of the VZ but not with post-mitotic GAD67+ cells in the overlying SVZ and mantle zone (Figure 3F). Thus pyramidal cells and interneurons show a reverse pattern of expression of *CHNRA4* and *CHNRB2*, with interneurons restricting expression to their progenitor cells, and pyramidal cells restricting expression to post-mitotic neurons.

## Discussion

This study provides further evidence that nAChRs may be present in the forebrain in the earliest stages of human fetal development. The majority of receptor subunits normally found in the brain were present at the mRNA level. Subunit combinations that would form functioning receptors were expressed, along with genes for proteins required to assemble the receptor at the cell membrane. In the cortex, gene expression was largely confined to post-mitotic glutamatergic neurons of the cortical plate and pre-subplate, or in the process of migrating to these locations. Cortical progenitor cells exhibited very low expression. Conversely, GABAergic interneurons showed low expression, but their progenitors in the ganglionic eminences exhibited higher expression.

### Presence of Ligands for nAChRs in the Developing Telencephalon

If functional nAChRs are to have active role in the development of the cortex it is necessary to show the presence of ligands to activate these receptors. Acetylcholine could derive from cholinergic innervation early in development but there is no evidence as yet for this occurring before about 12 PCW. From this age, choline acetyltransferase activity has been detected in cortex (Candy et al., 1985) although no earlier stage was tested, however, acetylcholinesterase histochemistry has only detected putative cholinergic fibers from the basal telencephalon entering the pre-subplate just before this stage of development (Kostovic, 1986). Synapses have been demonstrated to be present in the pre-subplate and marginal zone at this stage (Kostovic and Rakic, 1990; Bayatti et al., 2008). Whether these synapses include cholinergic terminals remains to be investigated. Our own attempts to demonstrate choline acetyltransferase immunoreactivity in paraffin sections of human fetal brain have so far been unsuccessful.

An alternative source could be choline and acetylcholine in the fetal circulation. Choline as a nutrient is critical for fetal nervous system development (Zeisel, 2006). High concentrations of choline are delivered to the fetus across the placenta by specialized transport systems (Sweiry et al., 1986). Choline can act as a ligand for α7 receptors in particular (Alkondon et al., 1997), however, these subunits were not found to be highly expressed in the present study. Choline appears to be a far less potent agonist for α4_2_β2_3_ receptors (Alkondon et al., 1997) the most likely isoform present in the developing cortex (see above), however, it can potentiate the action of acetylcholine on α4β4 receptors (Zwart and Vijverberg, 2000) as well as modulate synaptic transmission and neuronal excitability independent of nicotinic receptor activation (Albiñana et al., 2017). Furthermore, it has been demonstrated that the placenta can synthesize and store high concentrations of acetylcholine from as early as 9 PCW (Sastry et al., 1976) and release it into both maternal and fetal circulations (Sastry, 1997). Although recent evidence suggests an effective blood brain barrier has formed by this stage of development (Møllgård et al., 2017) it seems possible that acetylcholine may enter the developing brain and act in a paracrine manner.

### Potential Role for nACHRs in Development

It has long been appreciated that neurotransmitters can have paracrine effects in the developing cortex (Manent and Represa, 2007). Long before appreciable synaptogenesis has occurred there is release of GABA and glutamate by non-vesicular mechanisms that leads to communication between migrating neuronal pre-cursors in particular. It has been proposed that glutamate release by pyramidal cells attracts migrating inhibitory interneurons acting through ionotropic AMPA and NMDA receptors (Manent et al., 2005) and GABA released by interneurons influences pyramidal cell migration via ionotropic GABA_A_ receptors (Manent et al., 2006). In this way, a balanced mix of each neuronal subtype is achieved in the cortex (Manent and Represa, 2007). Intracellular Ca^2+^ concentration is an important coordinator of the intracellular processes controlling migration (Komuro and Kumada, 2005) and activation of all these ionotropic receptors can directly or indirectly lead to Ca^2+^ entering the neuron and increasing intracellular concentrations. Activation of nicotinic receptors should also lead to depolarization and the opening of voltage gated Ca^2+^ channels and NMDA receptors, raising intracellular Ca^2+^ (Kulbatskii et al., 2018). This study revealed nicotinic receptor subunits to be pre-dominantly expressed by post-mitotic glutamatergic neurons and not GABAergic neurons, suggesting their activation could preferentially control migration of one class of neuron only.

Acetylcholine can also stimulate proliferation of cancer cells via nicotinic receptors (Song et al., 2003 and Montaño-Velázquez et al., 2018) and its preferential expression by neural progenitors for GABAergic neurons rather than for glutamatergic neurons suggest that activation of nicotinic receptors may control GABAergic cortical interneuron production independently of production of glutamatergic neurons. Interestingly, cortical glutamatergic neuron progenitors preferentially express various glutamate receptor sub-units, whereas inhibitory interneuron progenitors do not (Bagasrawala et al., 2017) suggesting that the development of subsets of neurons is controlled by different neurotransmitters.

## Conclusion

The possibility that nicotinic receptors are expressed by different classes of post-mitotic neuron and neuroprogenitor cell in early human fetal forebrain raises the possibility that acetylcholine may act either as a neurotransmitter or paracrine agent to influence cortical development. Further *in vitro* experiments will be required to explore these possibilities. This raises the possibility that maternal ingestion of nicotine, either by smoking, or by oral or transcutaneous administration, may have effects upon brain development.

## Data Availability Statement

The datasets generated for this study can be found in the www.ebi.ac.uk/arrayexpress/experiments/E-MTAB-4840.

## Ethics Statement

The studies involving human participants were reviewed and approved by Newcastle and North Tyneside NHS Health Authority Joint Ethics Committee. The mothers provided their written informed consent to participate in this study.

## Author Contributions

GC and MS designed the study and directed the research. AA and WM carried out the research and collected the data. GC and AA analyzed the data and prepared the manuscript. MS and WM contributed to the final production of the manuscript.

## Conflict of Interest

The authors declare that the research was conducted in the absence of any commercial or financial relationships that could be construed as a potential conflict of interest.

## Footnotes

1 http://www.hdbr.org

## Abbreviations

KI67: cell cycle protein recognized by monoclonal antibody Ki67
RNAseq: sequencing of total RNA species isolated from tissues
GAD 67: glutamate decarboxylase isoform molecular weight 67 kD
*CHRNA4, CHRNB2, CHRNA5, CHNRA7, CHNRA1, CHNRB1*: Genes and mRNA for particular sub-units of the nicotinic receptor
GABA: gamma-aminobutyric acid.
